# *OsGIF1* Positively Regulates the Sizes of Stems, Leaves, and Grains in Rice

**DOI:** 10.3389/fpls.2017.01730

**Published:** 2017-10-05

**Authors:** Zhongshan He, Jing Zeng, Yun Ren, Dan Chen, Wenjie Li, Fengyan Gao, Ye Cao, Tao Luo, Guoqiang Yuan, Xianghong Wu, Yueyang Liang, Qiming Deng, Shiquan Wang, Aiping Zheng, Jun Zhu, Huainian Liu, Lingxia Wang, Ping Li, Shuangcheng Li

**Affiliations:** ^1^State Key Laboratory of Hybrid Rice, Sichuan Agricultural University, Chengdu, China; ^2^Rice Research Institute, Sichuan Agricultural University, Wenjiang, China; ^3^State Key Laboratory of Hybrid Rice, Hunan Hybrid Rice Research Center, Changsha, China

**Keywords:** rice, *OsGIF1*, organ size, cell size, knock-out

## Abstract

Growth-regulating factor (GRF) interacting factors (GIFs) are involved in several developmental processes in *Arabidopsis.* We previously showed that upregulation of *OsGIF1* expression improves rice grain size. However, whether *OsGIF1* is involved in other developmental processes remains unclear. Here, we report pleiotropic effects of *OsGIF1* on rice organ size regulation. Overexpression and functional knock-out via a CRISPR/Cas9 strategy revealed that *OsGIF1* not only positively regulates the sizes of rice leaf, stem, and grain but also influences rice reproduction. Expression profiles based on both qRT-PCR and *GUS* (*β-glucuronidase*) histochemical staining suggested that *OsGIF1* is differentially expressed across various rice tissues, consistent with its roles in regulating the development of multiple rice organs. Additionally, we found that OsGIF1-GFP localized preferentially in the nucleus, which supports its proposed role as a transcriptional cofactor. Further histological analysis suggested that *OsGIF1* affected rice organ size possibly by regulating cell size. Our results suggest that *OsGIF1* plays important roles in vegetative and reproductive developmental processes, with important implications for rice breeding.

## Introduction

Growth-regulating factor (GRF) interacting factor (GIF) was first reported as the major partner of the plant-specific transcription factor GRF (Kim and Kende, 2004; Horiguchi et al., 2005), which has been implicated in stem and leaf development (van der Knaap et al., 2000; Kim et al., 2003; Kim and Kende, 2004; Horiguchi et al., 2005; Omidbakhshfard et al., 2015). The amino acid (AA) sequences of GIFs are homologous to those of the human synovial translocation family protein SYT (Thaete et al., 1999; Kim and Kende, 2004; Horiguchi et al., 2005; de Bruijn and Geurts van Kessel, 2006; Kim and Tsukaya, 2015), which is also known as SS18. Both proteins lack the DNA binding domain (DBD) and act as transcriptional co-activators by interacting with human SWI/SNF ATPases (Brett et al., 1997; dos Santos et al., 1997; Thaete et al., 1999; Kato et al., 2002; Aizawa et al., 2004).

The SYT N-terminal homology (SNH) domain of a GIF is necessary because of its direct interaction with the QLQ domain of GRF in *Arabidopsis* and rice (Kim and Kende, 2004; Horiguchi et al., 2005; Liu et al., 2014; Duan et al., 2015; Li et al., 2016). Although GIF proteins do not have DBDs and NLSs, AtGIF1/AN3 protein is preferentially localized in the nucleus (Kim and Kende, 2004). Additionally, several pairs of GRF and GIF in *Arabidopsis* and rice have been demonstrated to work as complexes in the nucleus (Liang et al., 2014; Liu et al., 2014; Kim and Tsukaya, 2015). Multiple reports have tested the transactivation activities of AtGIF1/AN3 (Kim and Kende, 2004; Liu et al., 2014; Li et al., 2016). Three copies of GIFs have been annotated in the *Arabidopsis*, rice, and maize genomes, while nine, 12 and 17 GRF members have been found in *Arabidopsis*, rice and maize, respectively (Choi et al., 2004; Kim and Tsukaya, 2015; Omidbakhshfard et al., 2015). The interacting partnership between GRF and GIF, which is required for several developmental processes, has been confirmed in nearly all *Arabidopsis* members and has also been seen in rice (Kim and Tsukaya, 2015; Omidbakhshfard et al., 2015).

Growth-regulating factor interacting factor is involved in several vegetative and reproductive developmental processes in *Arabidopsis* (Kim and Tsukaya, 2015; Omidbakhshfard et al., 2015). In earlier studies, GIF was reported to be required in controlling cell proliferation during leaf development by interacting with GRF (Kim and Kende, 2004; Horiguchi et al., 2005). Interestingly, although the AN3/GIF1 transcripts are not detectable in leaf epidermal cells, the AN3/GIF1 protein can move into epidermal cells after being synthesized within mesophyll cells and helps to control epidermal cell proliferation (Kawade et al., 2013). The “compensation effect” phenomenon was also found in the *an3/gif1* mutant and further investigation demonstrated that the *an3*-dependent compensation was a non-cell autonomous process (Kim and Kende, 2004; Horiguchi et al., 2005; Kawade et al., 2010). GIF also works in adaxial/abaxial (Ad-Ab) patterning by the interaction of GIF1 with ASYMMETRIC LEAVES 2, a nuclear protein important for leaf Ad-Ab patterning (Iwakawa et al., 2002, 2007; Xu et al., 2003; Horiguchi et al., 2011). Moreover, *GIF* contributes to establishment of cotyledon identity by repressing the expression of an embryonic apical fate determination gene *PLETHORA1*, by cooperating with *HAN*, a GATA-type transcription factor (Kanei et al., 2012). In addition, GIF plays an important role in the determination of carpel number and male and female reproductive development in *Arabidopsis* and in husk/lemma development in rice, suggesting a role of *OsGIF* in floral organ determination and development (Lee et al., 2014; Liang et al., 2014; Liu et al., 2014; Duan et al., 2015; Li et al., 2016; Meng et al., 2016b). Interactions with numerous other proteins involved in chromatin remodeling processes, such as ATPases of the SWI/SNF family, have also been presented (Debernardi et al., 2014; Vercruyssen et al., 2014; Nelissen et al., 2015). Very recently, the AN3/GIF1-YODA cascade has been implicated in anthocyanin accumulation (Meng et al., 2016a), water-use efficiency and drought tolerance (Meng and Yao, 2015) in *Arabidopsis*. However, the function of rice GIF members remains ambiguous.

Plant organ size is a complex trait and is determined majorly by the process of cell proliferation and cell expansion. Significant progresses in dissecting of genetic factors that control plant organ size have been achieved in *Arabidopsis*. Several pathways, such as plant hormones, ubiquitination degredations, cytochrome P450 pathway and microRNAs, are implicated in this developmental process. Among which, *ERBB-3 BINDING PROTEIN 1* (Gingras et al., 2001), *AUXIN-REGULATED GENE INVOLVED IN ORGAN SIZE* (Hu et al., 2003) and *AINTEGUMENTA* (Elliott et al., 1996) were auxin related positive regulators for cell proliferation; The A-type and B-type *ARABIDOPSIS RESPONSE REGULATORS* could transmit the CK signal to the downstream gene for the promotion of cell proliferation (Dello Ioio et al., 2007). The *BIN2* and *BES1/BZR1* genes were key factors in the BR signaling pathway and positively regulate cell expansion (Halliday, 2004). While the *GIBBERELLIN INSENSITIVE* (Ubeda-Tomas et al., 2009) and *REPERSSOR OF GA1-3* (Silverstone et al., 2001) genes in GA signaling negatively regulated cell expansion. Besides, BIG BROTHER (Disch et al., 2006), an ubiquitin protein ligase, and DA1 (Li et al., 2008), an ubiquitin receptor, were both negatively regulators for cell proliferation, while *KLUH* (Anastasiou et al., 2007) and *EOD3* (Fang et al., 2012), both encoded cytochrome P450 monooxygenases, positively regulated cell proliferation. Moreover, miR396 was reported to negatively regulate cell proliferation by target degradation of several GRF members in *Arabidopsis* (Rodriguez et al., 2010), and the OsmiR396-GRF4-GIF1 regulatory module was demonstrated affecting rice grain size by influencing the cell size via simultaneously regulating the BR and GA pathways (Che et al., 2015; Duan et al., 2015; Li et al., 2016).

We previously found that OsGIF1 interacts directly with OsGRF4 and its upregulation improves rice grain size (Li et al., 2016). Thus, *OsGIF1* plays a role in regulating rice grain size. However, further analysis found that *OsGIF1* expression was not restricted to the spikelet, which might suggest that *OsGIF1* is involved in other developmental process. Here, we report the pleiotropic effects of *OsGIF1* on rice organ size regulation by analyzing the overexpression and functional knock-out (KO) of *OsGIF1* in rice. *OsGIF1* not only positively regulated the sizes of rice leaf, stem, and grain but also affected the rice reproductive process. The results suggest that *OsGIF1* plays an important role in vegetative and reproductive developmental processes, which has implications for rice breeding.

## Materials and Methods

### Plant Materials and Growth Conditions

Three KO lines and three overexpression lines were used in this study. The three overexpression lines, in which the *OsGIF1* (LOC_Os03g52320) was driven by the 2x35S promoter (Mao et al., 2005), were obtained by self-pollinating the T0 plants described in our previous report (Li et al., 2016). The japonica variety Nipponbare (Nipp) was used as control. All plants were planted in the experimental field at the Rice Research Institute, Sichuan Agricultural University, Wenjiang. Phenotypic data were collected at the maturing stage. The data were analyzed using Excel (Microsoft) for mean values and standard errors of mean (SEM). Statistical significance was assessed by conducting Student’s *t*-test.

### CRISPR/Cas9-Mediated KO of *OsGIF1*

The primer sequences for molecular cloning and constructions are listed in Supplementary Table 1. We verified *OsGIF1* function by generating two gRNA constructs, in which the gRNA was driven by the rice U6 promoter, and the plant-optimized Cas9 was driven by the UBI promoter (Miao et al., 2013). These constructs were introduced into the WT (Nipp) (Hiei et al., 1997). Then, the transgenic plants were subjected to PCR and sequencing analysis to determine the occurrence of mutations. To verify the association between the mutation in *OsGIF1* and the mutant phenotype, we performed segregation analysis in some populations generated by back-crossing these mutants with WT and confirmed the co-segregation of the mutant phenotype and mutations. To evaluate off-target effects, four putative off-target sites were identified by similar sequence searches within the rice genome (Supplementary Table 2). These sites were then sequenced for mutation analysis.

### Morphological and Cellular Analyses

The grain length, width, and 1,000-grain weight were measured by an automatic seed-size analyzing system (SC-G, Wanshen, Hangzhou). Other traits were investigated using conventional methods at maturing stage. An environmental scanning electronic microscope (QUANTA 450, Nikon) was employed to observe the outer surface of the leaf, stem internode, and outer glume. For histological analysis, samples of the leaf and stem internode were placed in the FAA solution for 12 h at 4°C, dehydrated in a graded ethanol series, followed by substitution using 3-methylbutyl acetate (Li et al., 2016). The samples were dissected and observed under a microscope (80I, Nikon) for determinations of cell number and size.

### GUS Staining Assay

To localize the transcripts of *OsGIF1* in rice tissue, a *OsGIF1*pro::*GUS* construct in which expression of the *GUS* gene was driven by native promoters was generated. Then, the *OsGIF1*pro::*GUS* construct was introduced into the *Agrobacterium tumefaciens* strain EHA105 to transform WT plants. Histochemical GUS staining was performed in the transgenic plants as described previously (Li et al., 2013).

### qRT-PCR Analysis

Total RNAs were isolated from various rice tissues at different developmental stages using the TriPure Isolation reagent (Roche). The cDNAs were then reverse-transcribed using the Transcriptor First-Strand cDNA Synthesis kit (Roche). qRT-PCR was conducted in a total volume of 10 μl, with 0.3 μl of the reverse-transcribed product, 0.08 mM gene-specific primers, and 5.0 ml of Sso Advanced TM SYBR Green Supermix (Bio-Rad) using a Bio-Rad CFX96 Real-Time PCR System according to the manufacturer’s instructions. Data were analyzed by the relative quantification method (Livak and Schmittgen, 2001). The rice *Actin* gene was used as internal control. Each measurement was determined for at least two biological samples using three replicates for each sample.

### Subcellular Localization of OsGIF1

The full-length cDNA of *OsGIF1* was cloned into the pA7-GFP vector to generate the 2x35S::OsGIF1-GFP cassette. This plasmid was then introduced into rice protoplast cells for transient expression (Li et al., 2013). GFP signals were visualized using a confocal scanning microscope (Nikon A1, Kanagawa, Japan) 24–48 h after transformation.

## Results

### KO of *OsGIF1* in a CRISPR/Cas9 Strategy

**FIGURE 1:**
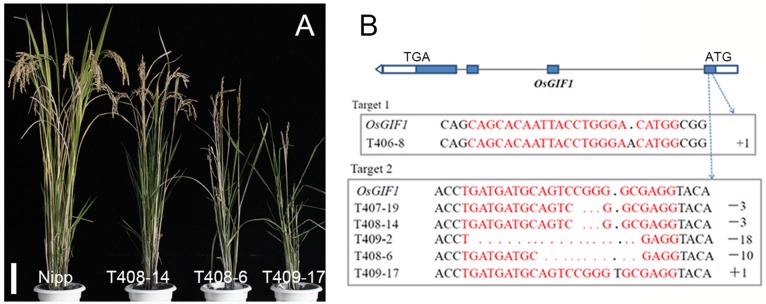
Knock-out (KO) of *OsGIF1* by a CRISPR/Cas9 strategy. **(A)** Comparisons of the *OsGIF1* KO plants, scale bar: 10 cm; **(B)** Identification of mutation by direct sequencing of target sites in transgenic plants.

In order to investigate the function of *OsGIF1*, plasmids containing CRISPR-Cas9 guide RNAs (gRNAs) against two target sites within the first exon of *OsGIF1* were made (**Figure 1B**). The plasmids were then transformed into the wild-type (WT) variety of Nipponbare (Nipp), and approximately 30 transgenic plants were obtained for each plasmid. Sequencing of PCR-amplified *OsGIF1* genomic DNA from transgenic plants showed five types of homozygous mutations within the target sites: one in target site 1 (an A insertion) and four in target site 2 (3-base deletions, 18-base deletions, 10-base deletions, and an T insertion) (**Figure 1B**). Although no biallelic plants were obtained, several heterozygous transgenic plants of the five mutations were found, and no phenotypes were observed.

Interestingly, the phenotype of the homozygous *osgif1* mutant differed from its mutation type. Slight effects were observed on plant height in the 3-/18-base-deletion plants. By contrast, the A/T insertion plants had severe effects on plant height, leaf development, and panicle construction. Additionally, the 10-base-deletion plants had moderate impact on these traits, compared with plants bearing the other two mutations (**Figures 1A,B**). These phenotypic differences are to some extent in accordance with the effects of each mutation on the protein sequence of OsGIF1. The 3-/18-base deletions only deleted 1 or 6 AAs without changing the coding frame of OsGIF1, while the A/T insertion and 10-base deletions resulted in evidently truncated OsGIF1 by introducing premature stop codons (Supplementary Figure 1). To elucidate whether these differences in phenotype were also linked with off-target effects of the gRNAs, we sequenced several potential off-target sites and found no off-target editing for both gRNAs (Supplementary Table 2). Further linkage analysis of a F2 populations (28 plants in total) generated by the crosses of the T408-6 mutants and the WT found that all the plants with homozygotic mutations were showed in the mutant phenotype (6 plants), whereas other plants carrying no (10 plants) or heterozygous mutations (12 plants) were all normal in phenotype, confirming the association between the mutation and the mutant phenotype. Thus, the phenotype was directly caused by *OsGIF1* mutation, and the phenotypic diversity of the different mutation types might have resulted from varying degrees loss of *OsGIF1* function.

### *OsGIF1* KO Has Pleiotropic Effects on Rice Development

**FIGURE 2:**
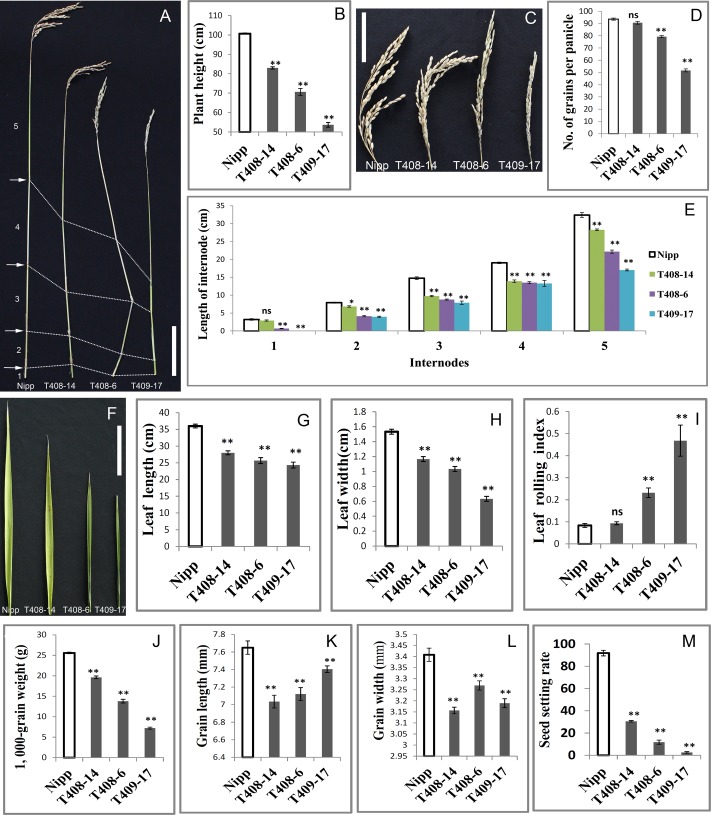
Pleiotropic effects of *OsGIF1* KO on rice development. Comparisons of several important traits between the *OsGIF1* KO and control plants. **(A)** Stem; **(B)** plant height; **(C)** panicle; **(D)** number of grains per panicle; **(E)** internode length; **(F)** leaf; **(G)** flag leaf length; **(H)** flag leaf width; **(I)** leaf rolling index; leaf rolling index = (*L*w – *L*n)/*L*w; *L*w, distance of leaf blade margins at unfolding state; *L*n, distance of leaf blade margins at natural state; **(J)** 1000-grain weight; **(K)** grain length; **(L)** grain width; and **(M)** seed setting rate. Scale bars: 10 cm. Values are all shown as means ± standard error of the mean (SEM) (*n* = 3). Asterisks indicate significant differences between the WT and KO plants as determined by Student’s *t*-test: ns, not significant; ^∗∗^*p* ≤ 0.01; ^∗^*p* ≤ 0.05.

The most noticeable phenotype in the *OsGIF1* KO plants was reduction of plant height. Three types of mutant phenotypes were found (**Figure 1A**). Thus, we selected three representative mutant plants, namely, T408-14, T408-6, and T409-17 to analyze the phenotype and function of *OsGIF1*. The plant height of all the KO plants decreased highly significantly compared with that of the WT (**Figures 2A,B**). Consistent with decrease in height, most of the mutant panicles became smaller, and the number of grains per panicle in the mutants was reduced (**Figures 2C,D**). We also examined the stems and internodes of mutant plants, since rice plant height is primarily determined by internode length of the stem. The results showed that almost all mutant stems and their internodes were considerably shortened, compared with their WT counterparts (**Figures 2A,E**). These results demonstrated that functional loss of *OsGIF1* led to reduced plant height of rice by shortening the internode length of stems.

*OsGIF1* KO plants also shows phenotypes related to leaf development (**Figure 2F**). Leaf lengths and widths of all the mutant plants were significantly reduced compared with those of the WT (**Figures 2G,H**). Besides, these mutants also showed different degrees of leaf rolling. The leaf rolling indices of plants with the most severe mutations, namely T408-6 and T409-17, were sharply increased, with almost all leaves rolled compared with those of the WT (**Figures 2F,I**). These results indicated that *OsGIF1* affected rice leaf development by regulating both leaf size and leaf rolling.

Rice is an important crop as a source of carbohydrate for more than half of the world population. Therefore, we investigated whether *OsGIF1* affects the grain yield of rice. We found that grain weights of all mutant plants were significantly decreased compared with that of the WT (**Figure 2J** and Supplementary Figure 2). The reduced grain weights were further observed to be mainly caused by significant decreases in grain length and grain width of the *OsGIF1* KO plants (**Figures 2K,L**). This indicated that *OsGIF1* also functioned in rice seed development, which is consistent with our previous findings (Li et al., 2016).

Reductions in the panicle seed setting rate were also observed in the *OsGIF1* KO plants (**Figure 2M**), suggesting a role of *OsGIF1* in regulating the rice reproduction process. The degree of reduction was consistent with the mutant type. In the most severely mutated plant, T409-17, the seed setting rate was extremely low, and the plant became almost completely sterile (**Figure 2M**). The floral structures in T409-17 at the heading date manifested the following series of abnormalities in several whirls of floral organs: tightly wrapped spikelets, shorter, twisted, or ectopic paleas/lemmas, decreased stamens, increased pistils, and white-colored sterile anthers (Supplementary Figure 2). These results indicated that serious KO of *OsGIF1* led to various floral organ abnormalities and apparent reductions in the seed setting rate, suggesting an important role of *OsGIF1* in the determination of rice floral organs.

### Overexpression of *OsGIF1* Increased the Size of Multiple Rice Organs

**FIGURE 3:**
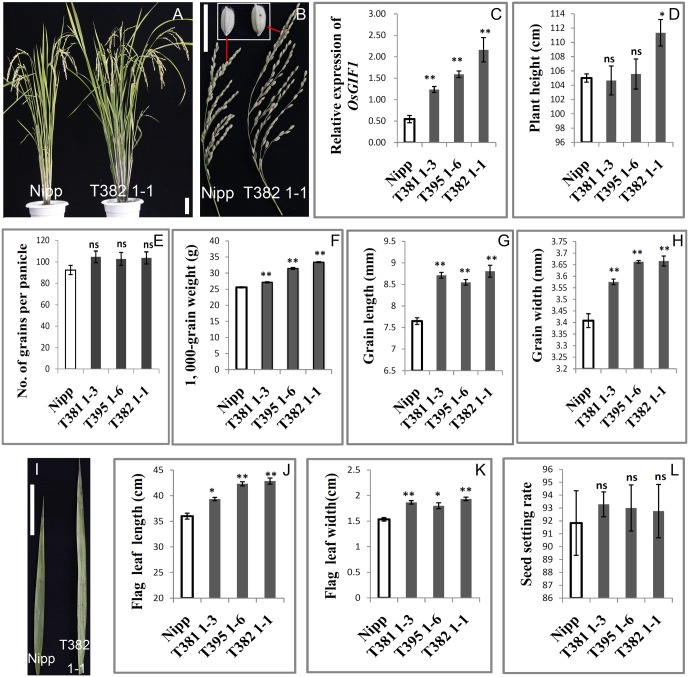
Increase in the size of multiple rice organs caused by overexpression of *OsGIF1.* Comparisons of several important traits between the *OsGIF1* overexpression and control plants. **(A)** Plant phenotype; **(B)** panicle and grain; **(C)** relative expression level of *OsGIF1*; **(D)** plant height; **(E)** number of grains per panicle; **(F)** 1000-grain weight; **(G)** grain length; **(H)** grain width; **(I)** leaf; **(J)** flag leaf length; **(K)** flag leaf width; and **(L)** seed setting rate. Scale bars: 10 cm. Values are all shown as mean ± SEM (*n* = 3). Asterisks indicate significant differences between the WT and overexpression plants as determined by Student’s *t*-test: ns, not significant; ^∗∗^*p* ≤ 0.01; ^∗^*p* ≤ 0.05.

We performed detailed investigations of multiple organs of several representative overexpression plants designated as T381 1-3, T395 1-6, and T382 1-1. First, qRT-PCR was performed to confirm the overexpression of *OsGIF1* within these representative plants (**Figure 3C**). The plant heights of the overexpressing plants were slightly or sometimes significantly increased (**Figures 3A,D**). The transgenic plants also exhibited larger panicles than the WT (**Figure 3B**) although the number of grains per main panicle did not differ significantly between the transgenic and WT plants (**Figures 3E,L**). Consistent with our previous work (Li et al., 2016), overexpression of *OsGIF1* in these plants also significantly increased grain size and weight by synchronously increasing grain length and width (**Figures 3B,F–H**). Additionally, overexpression of *OsGIF1* apparently affected leaf development, because all of these plants exhibited highly significantly increased leaf length and width (**Figures 3I–K**). These results confirmed that overexpression of *OsGIF1* increased the size of multiple rice organs, indicating a positive role of *OsGIF1* in regulating rice organ size.

### *OsGIF1* Controls Rice Organ Size Possibly by Regulating Cell Expansion

**FIGURE 4:**
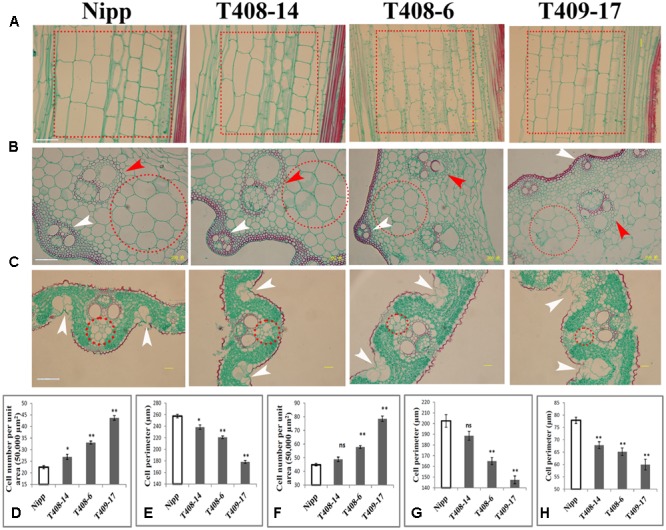
*OsGIF1* regulation of cell size to control rice organ size. **(A–C)** Paraffin section analysis of the stems and leaves of KO plants. **(A)** Longitudinal section of stem internode; dotted boxes in red indicate the major cell types selected for cell counting and measuring. **(B)** Cross section of stem internode. Red arrow heads indicate inner vascular bundles, and white arrow heads indicate outer vascular bundles. Dotted circles in red indicate the major cell types selected for cell counting and measuring. **(C)** Cross section of leaves. White arrow heads indicate bulliform cells. Dotted circles in red indicate the major cell types selected for cell counting and measurement. The first internode from the bottom and the middle region of the flag leaf of all plants were collected for observation. Scale bars (50 μm) are shown in Nipp. **(D–H)** Cell number and size determinations. **(D)** Cell number per unit area in **A**; **(E)**, cell size in **A**; **(F)**, cell number per unit area in **B**; **(G)**, cell size in **B**; **(H)**, cell size in **C**. For cell numbers (per unit area) counting, a total area of 50,000 μm^2^ for each sample was investigated. For cell volume determination, 10 representative cells within the dotted circles/boxes regions were selected for cell size measuring. Values are all shown as mean ± SEM (*n* = 3). Asterisks indicate significant differences between the WT and KO plants as determined by Student’s *t*-test: ns, not significant; ^∗∗^*p* ≤ 0.01; ^∗^*p* ≤ 0.05.

To investigate the cellular effect of *OsGIF1*, we first performed paraffin sectioning of stem internodes of these plants. Longitudinal histological sectioning analysis showed a clear trend toward reduced cell volume in the KO plants, especially in the severe KO plants (**Figure 4A**). Overall, these results are consistent with the degree of organ size alternation in these plants (**Figure 1A**). To further address this, we then counted cell numbers per unit area and found significant increases in T408-14 (+19.92%), T408-6 (+47.39%), and T409-17 (+94.8%) when compared to WT (**Figure 4D**), suggesting that cell sizes of the KO plants were altered. Direct cell measurement showed that the cell perimeters were significantly reduced in T408-14 (-7.39%), T408-6 (-16.6%) and T409-17 (-30.77%) (**Figure 4E**). Transverse sectioning of these internodes (**Figure 4B**) further confirmed the significantly increased cell numbers per unit area (+28.86% in T408-6 and +75.03% in T409-17) (**Figure 4F**) and reduced cell size (-18.57% in T408-6 and -27.23% in T409-17) (**Figure 4G**) in the stems of severe mutants. Consistent with the result of stems, transverse section of flag leaves (**Figure 4C**) also showed significantly reduced cell size in T408-14 (-12.93%), T408-6 (-16.33%), and T409-17 (-23.06%) (**Figure 4H**). Since grain size is influenced by spikelet hull, we next performed scanning electron microscopy (SEM) analysis of the spikelet hulls of the overexpression and KO plants. Results showed that T382 1-1 exhibited significantly enlarged cell volume compared to WT, while T409-17 showed opposite effect of reduced outer glume cells (Supplementary Figure 3). Cellular measurement further showed that, compared with those in the WT, the length and width of epidermal cells of the outer glumes increased by 43.8 and 15.22% in T382 1-1, and decreased by 16.64 and 12.18% in T409-17, respectively (Supplementary Figure 3). Taken together, these results suggest that *OsGIF1* enhances the size of multiple important rice organs predominantly by promoting cell enlargement.

We also observed other morphological abnormalities except for cell size alternation in these plants. Transverse section of internodes revealed that layers of sclerenchyma cells of the stem vascular bundle and the number and size of the stem vascular bundle were apparently decreased in the KO plants (**Figure 4B**). Development of the stem vascular bundle was also affected, with incomplete development of outer vascular bundle occurring in the most serious KO plants (**Figure 4B**). Besides, SEM analysis of flag leaf revealed that the morphologies and organization patterns of cells were also altered in T382 1-1 and T409-17 (Supplementary Figure 4). Stoma guard cells are typically arranged in the longitudinal direction and separated by a longitudinal line of dumbbell silicon cells in the WT. However, two longitudinal lines of dumbbell silicon cells and several incompletely developed lines of stoma guard cells were observed both in T382 1-1 and T409-17. Several cells from these regions were morphologically abnormal compared to the WT. Interestingly, the number of silicon papillae on the leaf stoma guard cells seemed to have increased both in the overexpressing and KO plants (Supplementary Figure 4). Moreover, compared to the WT, organization patterns of stem exterior cells were slightly changed both in overexpressing and KO plants. However, abnormal development of stoma guard cells was especially apparent in T409-17, with significantly fewer stoma guard cells in the stem (Supplementary Figure 4), which is consistent with the findings of a recent study in which a role of AN3/GIF1 in modulating stomatal density was reported (Meng and Yao, 2015). Notably, the number of bulliform cells in the leaves of the serious KO plants was increased (**Figure 4C**), which abolished the balance of the Ad-Ab patterning and consequently led to rolled leaves. Thus, our findings reveal a cellular mechanism of the leaf-rolling phenotype in the most severe KO plants (**Figures 2F,I**), consistent with the function of SEMI-ROLLED LEAF1, a gene that modulates rice leaf rolling by regulating the formation of bulliform cells (Xiang et al., 2012). These results suggest that *OsGIF1* might also affect other cellular processes during rice organ or tissue development.

### Expression Profile of OsGIF1

**FIGURE 5:**
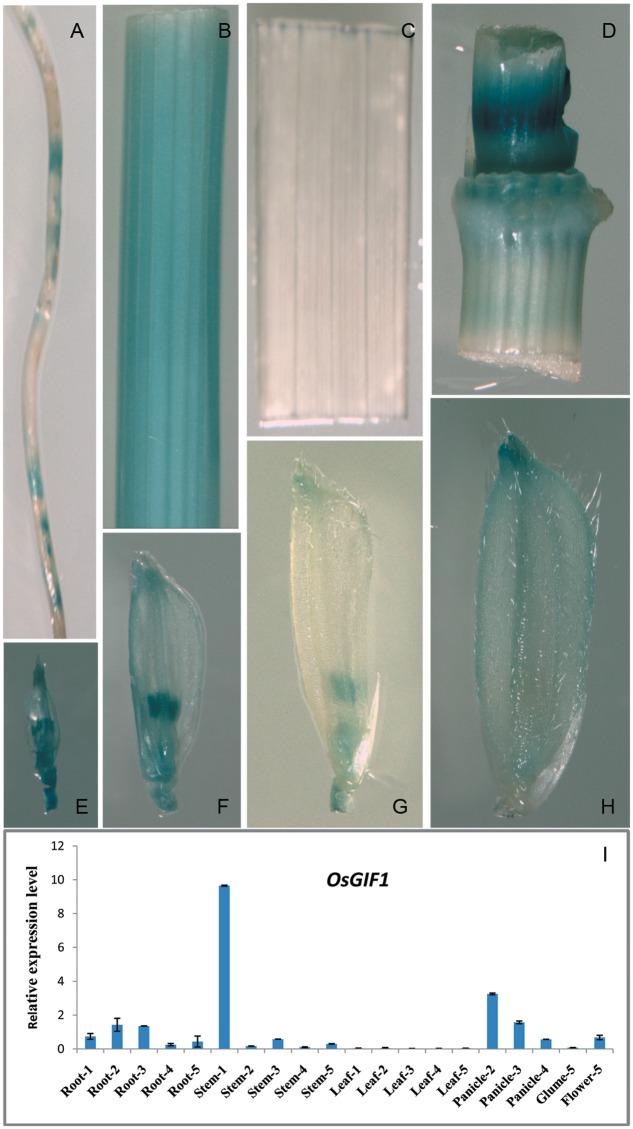
Expression profile of *OsGIF1.* **(A–H)** GUS staining of various tissues of *OsGIF1*pro::*GUS* transgenic plants. **(A)** Root; **(B)** internode; **(C)** leaf; **(D)** stem node; **(E–H)** Spikelets of plants with panicle lengths of 2 **(E)**, 5 **(F)**, 10 **(G)**, and 15 cm **(H)**, respectively. **(I)** Relative expression levels of *OsGIF1* in various tissues of WT plants. (1), (2), (3), (4), and (5) indicate plants with panicle lengths of 0, 2, 5, 10, and 15 cm, respectively. Values are all shown as mean ± SEM (*n* = 3).

We generated a construct in which the *GUS* gene was driven by the approximately 1.8 kb *OsGIF1* promoter to examine the temporal and spatial expression patterns of *OsGIF1*. Histochemical staining suggested that *OsGIF1* was differentially expressed in various rice tissues. Expression was relatively weak in the root (**Figure 5A**) and mature glume (**Figure 5H**) but was strong in the internode (**Figure 5B**), node (**Figure 5D**), the developing spikelet (**Figures 5E–G**), and especially in the developing anther (**Figures 5E–G**). Results of qRT-PCR assays further confirmed this result by showing that *OsGIF1* was highly expressed in developing stems but moderately in the root, young panicle, and flower (**Figure 5I**). Overall, the expression pattern of *OsGIF1* is consistent with its function. However, both GUS staining and qRT-PCR analysis indicated extremely low expression of *OsGIF1* in leaves approaching maturity (**Figures 5C,I**). Taken together with our results that *OsGIF1* is involved in leaf development (**Figures 2F–I, 3I–K**), these findings point toward a role for *OsGIF1* in the early development of rice leaf.

**FIGURE 6:**
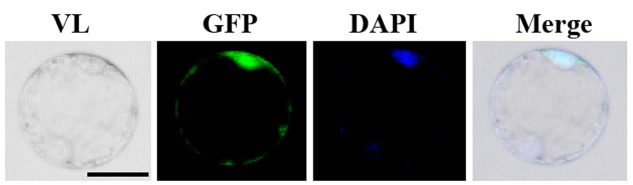
Subcellular localization of OsGIF1. Confocal images of rice protoplast cells after 48 h of infection. The OsGIF1 protein (visualized based on GFP signal) was mainly localized in the nucleus in rice cells transfected with the 2x35S::OsGIF1-GFP construct. Scale bar: 25 μm.

In order to investigate the subcellular localization of OsGIF1, we constructed an OsGIF1-GFP (green fluorescent protein) fusion construct with its expression driven by the CaMV 35S promoter. Transient expression in the rice protoplast cells showed that OsGIF1-GFP localized preferentially in the nucleus and was weakly expressed in the cytoplasm (**Figure 6**). This result is consistent with the idea that GIF proteins function as transcriptional co-activators by forming complexes with GRF transcription factors.

## Discussion

Many lines of evidence indicate that GIF genes are involved in regulating the vegetative growth of multiple organs (Iwakawa et al., 2002, 2007; Xu et al., 2003; Kim and Kende, 2004; Horiguchi et al., 2005, 2011; Kanei et al., 2012; Debernardi et al., 2014; Vercruyssen et al., 2014; Kim and Tsukaya, 2015; Nelissen et al., 2015; Omidbakhshfard et al., 2015), reproductive development (Lee et al., 2014; Liang et al., 2014; Meng et al., 2016b), and stress tolerance (Meng and Yao, 2015) in *Arabidopsis* plants. In the present study, KO of *OsGIF1* led to the following pleiotropic defects in rice development: reduction of plant height caused by significantly shortened internodes; decreased leaf size due to reduction in leaf length and width; and small seed size owing to reduction in grain length and width. The functions of *OsGIF1* were further elucidated by results of our overexpression studies. Rolled leaves and various kinds of floral organ abnormalities were also observed in the most severe KO of *OsGIF1*. Our results as a whole are in agreement with those reported previously in *Arabidopsis* and suggest various roles of *GIF1*/*OsGIF1* during plant organ development.

The most important function of *GIF1*/*OsGIF1* appears to be the determination of the size of different organs. However, the cellular effect of *OsGIF1* in rice may differ from its counterpart *GIF1* in *Arabidopsis*. Organ sizes of plants have been reported to be enlarged by two major mechanisms, namely, by promoting cell expansion or increasing cell proliferation. The majority of reports in *Arabidopsis* indicate that GIF1 influences organ size by promoting cell proliferation (Kim and Tsukaya, 2015; Omidbakhshfard et al., 2015). In the present study, we demonstrated that *OsGIF1* affects leaf size, length of the stem internodes, and seed size possibly by promoting cell expansion. Recently, a major QTL, namely *GLW2/GL2/GS2*, the allelic mutation of OsGRF4, was reported to positively regulate grain weight and size in rice by several independent groups (Che et al., 2015; Duan et al., 2015; Hu et al., 2015; Li et al., 2016). Further investigations demonstrated that OsGRF4 directly interacts with OsGIF1 to positively regulate seed size in rice mainly by promoting cell expansion (Che et al., 2015; Duan et al., 2015; Li et al., 2016)^.^ Although, the detailed cellular mechanism underlying this function of OsGRF4 remains unknown, the results are consistent with our findings that OsGIF1 might be involved in organ size regulation via mechanisms controlling cell expansion. The difference in *GIF1* functions between rice and *Arabidopsis* is very interesting but remains elusive due to a fundamental lack of understanding of functions of the rice GIF proteins and their possible partners. How and when this difference occurs should be clarified in future investigations.

However, our results also suggest a role of *OsGIF1* in regulating cell proliferation of some tissues, such as the leaf bulliform cells and silicon papillae of stoma guard cells in rice. Recent works in rice also suggest that the cell proliferation pathway contributes to grain size regulation, but is far less important than cell expansion (Che et al., 2015; Duan et al., 2015; Li et al., 2016). These results might also suggest that both cellular pathways work simultaneously in rice. Another difference in *GIF1* function between rice and *Arabidopsis* is that, although *GIFI* is involved in floral organ development, and despite differences in their mutant phenotypes, only the *Arabidopsis gif1 gif2 gif3* triple mutant, but not the single or double mutants showed obvious phenotypes in reproductive organs (Lee et al., 2014). By contrast, in rice, severe KO of *OsGIF1* single gene produced several reproductive organ abnormalities. These results suggest real functional differences between *OsGIF1* and *GIF*.

Interestingly, Che et al. (2015) demonstrated that OsGRF4/GL2 functions by activating brassinosteroid response, and simultaneously, the elevated BR response stimulates cell elongation by promoting gibberellin (GA) biosynthesis in rice seedlings. Consistently, in the present study, we showed that *OsGIF1* affects the size of leaf, stem internodes, and seed majorly by promoting cell expansion. Considering that GIFs have been widely proved to function in complexes with GRFs in *Arabidopsis* and rice (Kim and Tsukaya, 2015; Omidbakhshfard et al., 2015), it is reasonable that the similar biochemical pathways, like the BR and GA pathways, may also influence the leaf/stem cell size in rice. However, several other direct evidences are necessarily needed to apply the OsmiR396-GRF4-GIF1 regulatory module and its related biochemical mechanism to other organs.

Sequencing of *OsGIF1* KO plants revealed five types of homozygous mutations within the target sites. The phenotype of the homozygous *osgif1* mutant differed from its mutation type as follows: 3-/18-base deletion (resulted in 1 or 6 AA deletions) plants showed slight effects; A/T insertion (resulting in premature TGA stop codon at the 173rd AA) plants exhibited the most severe phenotypes; and the 10-base-deletion plants (resulting in premature TGA stop codon at 101st AA) have moderate effects. The range of mutant phenotypes was not caused by off-target effects of the gRNAs and might have resulted instead from varying degrees of loss of *OsGIF1* function. The phenotypic difference between the 3-/18-base-deletion and A/T insertion plants suggest that the 101–226 AAs polypeptide segment of OsGIF1 has an important function in regulating rice organ size. However, phenotypic differences between 10-base-deletion and A/T insertion plants suggest that the new 101–173 AAs polypeptide segment produced by A/T insertion frameshift mutation might have antagonistic effects against the residual function of the truncated OsGIF1 protein (1–101 AAs). Although, the major functions of GIF are being gradually elucidated in plants, further efforts are necessary to gain a deeper understanding of the detailed molecular function of each polypeptide fragment of OsGIF1.

## Author Contributions

SL and PL designed the experiments and directed the project; JiZ and ZH performed the cloning and functional analysis and collected almost all of the data; YR and FG performed the expression analysis and tissue localization and cell localization; DC, WL, and YC performed genetic transformations; TL, GY, and XW performed the phenotypic characterization of the mutant and transgenic plants; SW, HL, LW, and QD carried out the field experiments and investigations; YL, JuZ, and AZ constructed all the vectors; SL analyzed the data and wrote the paper.

## Conflict of Interest Statement

The authors declare that the research was conducted in the absence of any commercial or financial relationships that could be construed as a potential conflict of interest.
