# Supplementary material for: OsGIF1 Positively Regulates the Sizes of Stems, Leaves, and Grains in Rice
Source: Front Plant Sci. 2017 Oct 5;8:1730. doi: 10.3389/fpls.2017.01730 (PMC5633614; doi:10.3389/fpls.2017.01730)
Supplement: Supplementary file 1 [file Presentation_1.pdf]

OsGIF1 Positively Regulates the Sizes of Stems, Leaves and Grains in Rice

Zhongshan He[1,2#], Jing Zeng[1,2#], Yun Ren[1,2], Dan Chen[1,2], Wenjie Li[1,2], Fengyan Gao[1,2], Ye Cao[1,2], Tao Luo[1,2], Guoqiang Yuan[1,2], Xianghong Wu[1,2], Yueyang Liang[1,2], Qiming Deng[1,2], Shiquan Wang[1,2], Aiping Zheng[1,2], Jun Zhu[1,2], Huainian Liu[1,2], Lingxia Wang[1,2], Ping Li[1,2,3*], Shuangcheng Li[1,2*]

# These two authors contribute equally to this work

* Corresponding author: Ping Li (liping6575@163.com) and Shuangcheng Li (lisc926105@163.com)

[1] State Key Laboratory of Hybrid Rice, Sichuan Agricultural University, Chengdu 611130, China;

[2] Rice Research Institute, Sichuan Agricultural University, 211 Huimin Road, Wenjiang 611130, Sichuan, China;

[3] State Key Laboratory of Hybrid Rice, Hunan Hybrid Rice Research Center, Changsha 410125, China.

1 Supplementary Figures and Tables

1.1 Supplementary Figures

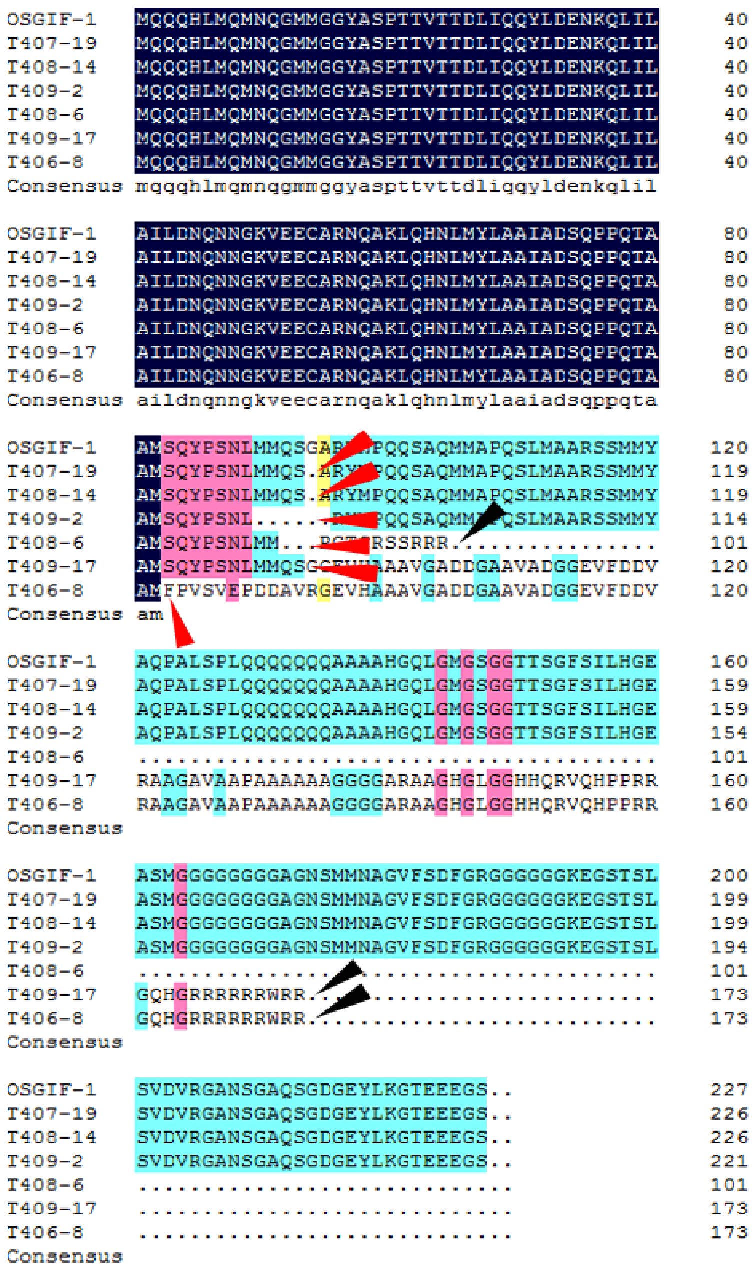

Supplementary Figure 1. Putative amino acid sequence alignments across all mutants.

A red arrow head indicates the target mutation, and a black arrow head indicates premature stop codon caused by the target mutation. Different background colors indicate different identities of multiple sequence alignments.

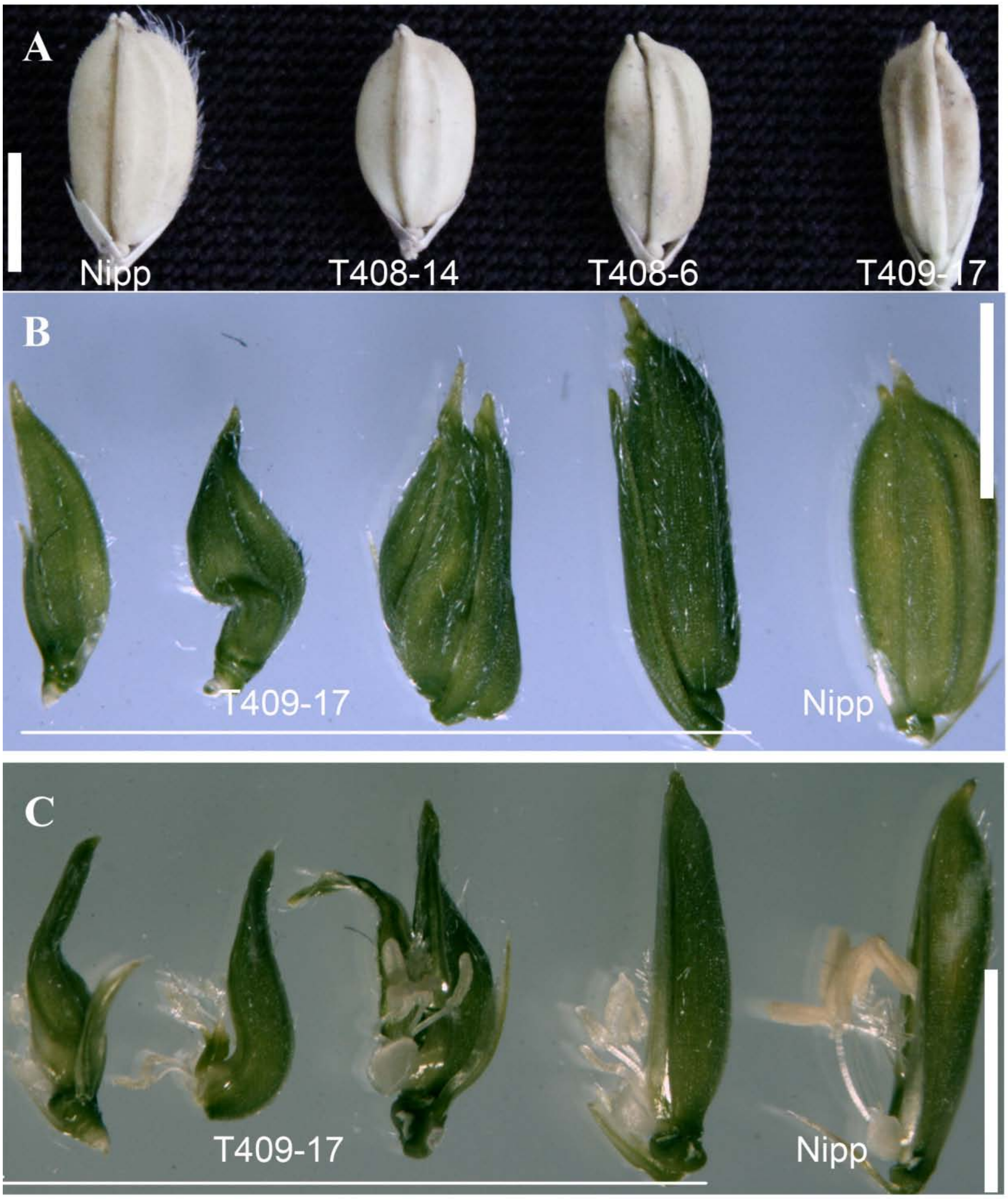

Supplementary Figure 2. Grain and floral organ observations in KO mutants.

(A) grain; (B) spikelet; (C) floral structure. Scale bar, 5mm.

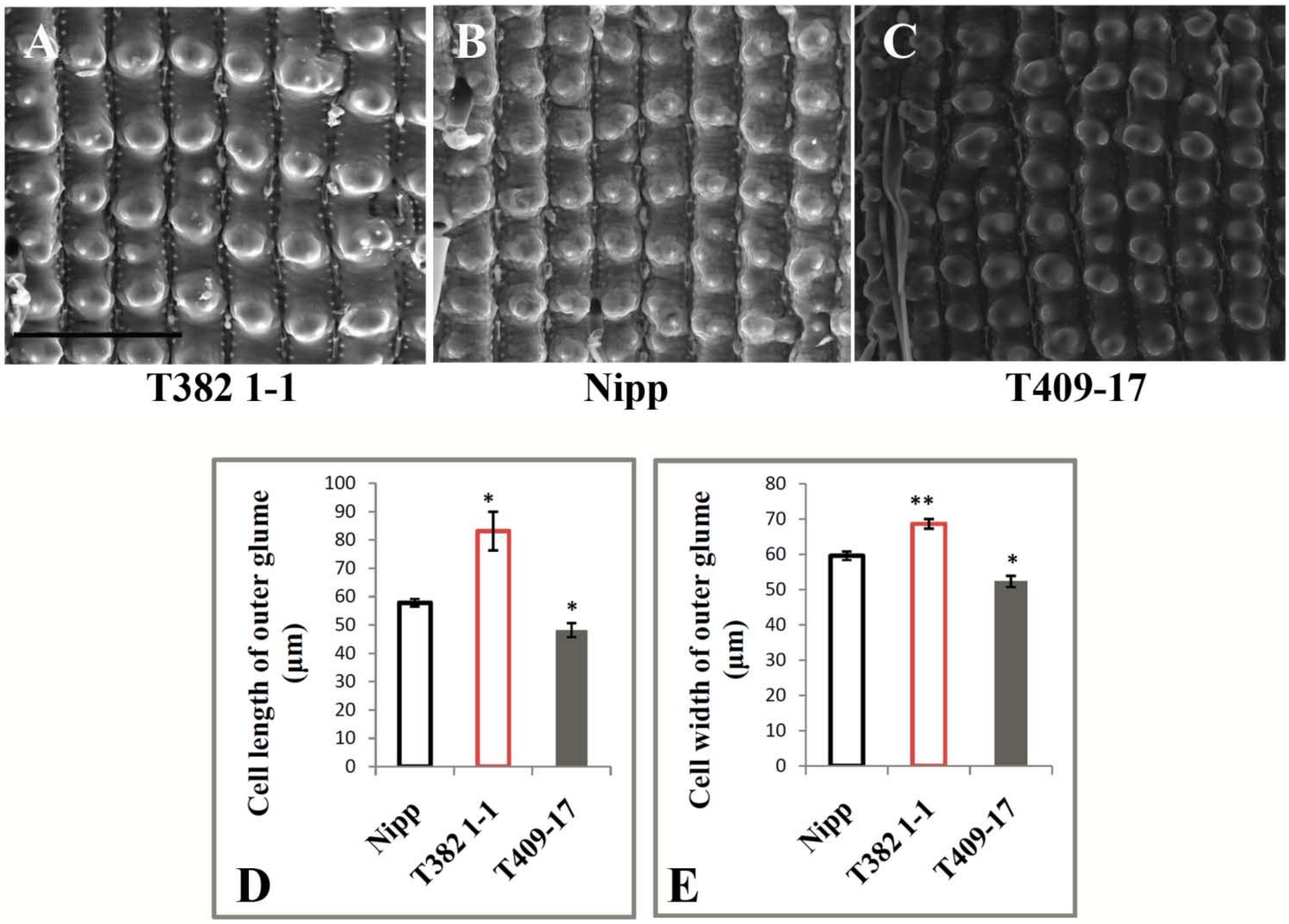

Supplementary Figure 3. SEM analysis of the outer glumes in KO and overexpression plants.

(A-C), SEM analysis of the outer glumes in KO and overexpression plants; Scale bar, 200 µm; (D), cell length of the outer glume; (E), cell width of the outer glume. Scale bar, 200 µm. Values are all shown as mean ± s.e.m (n=3). Asterisks indicate significant differences between the WT and overexpression/KO plants as determined by Student's *t*-test: ns, not significant; **, $p\leq 0.01$; *, $p\leq 0.05$.

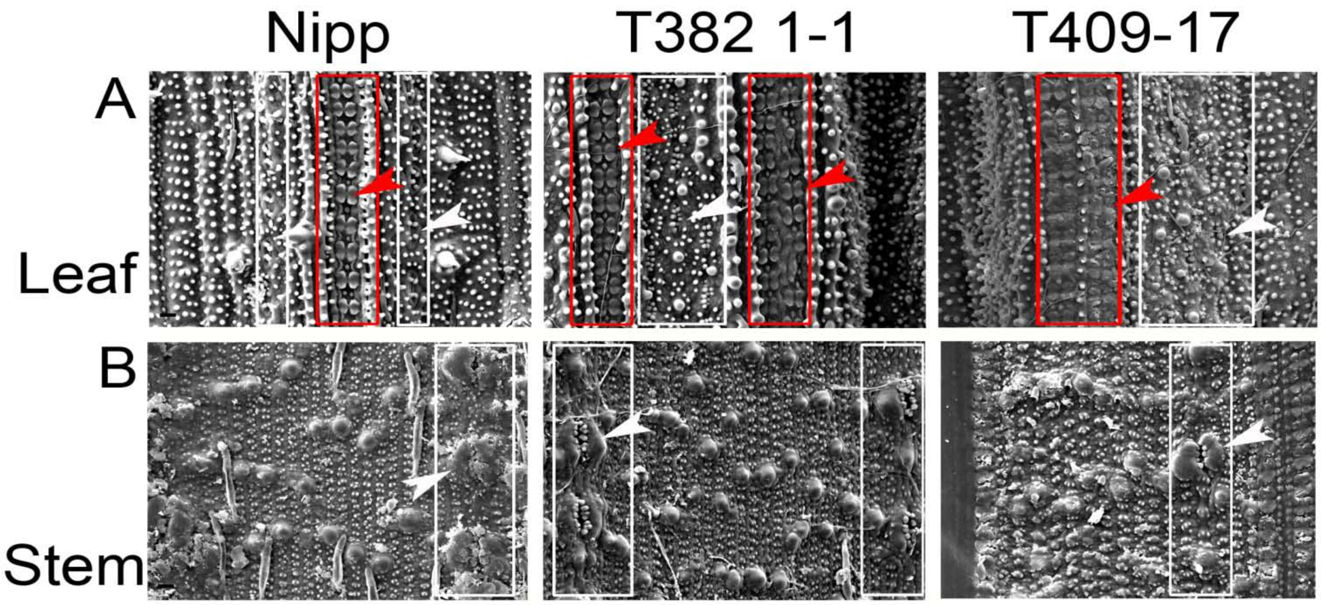

Supplementary Figure 4. SEM analysis of the outer surfaces of leaves and stems of KO and overexpression plants.

(A) SEM analysis of the outer surfaces of leaves. (B) SEM analysis of the outer surfaces of stems. Dotted boxes in red indicate the longitudinal array of dumbbell silicon cells, and dotted boxs in white indicate longitudinal array of stoma guard cells. Red arrow heads indicate dumbbell silicon cells, and white arrow heads indicate stoma guard cells. Scale bars, 10 µm.

1.2 Supplementary Tables

Supplementary Table 1. Primers used in this study

Primer name	Sequence (5’-3’)	Purpose
GIF1C-F	GCCGGATCCATGCAGCAGCAACACCTG	GIF1-cDNA,Transgenic construct
GIF1C-R	GGACTAGTGCTGCCTTCCTCCTCGGTG	GIF1-cDNA,Transgenic construct
PGIF1-GUS-F	ACGCGTCGACGAGAGGAGTCCGACCTCAAT	Promoter analysis, Transgenic construct
PGIF1-GUS-R	ACGCGTCGACTCCAATCAAAAACCTTTATAC	Promoter analysis, Transgenic construct
GIF1-GFP-F	GCCGGATCCATGCAGCAGCAACACCTG	GFP, Subcellular localization
GIF1-GFP-R	GGACTAGTGCTGCCTTCCTCCTCGGTG	GFP, Subcellular localization
qGIF1-F	TGCTTCCCCTACCACCGTC	q-RT-PCR
qGIF1-R	CGAGCGCACTCTTCCACCT	q-RT-PCR
Actin-F	CTTCATAGGAATGGAAGCTGCGGGT	Internal reference
Actin-R	CGACCACCTTGATCTTCATGCTGCTA	Internal reference

Supplementary Table 2. Information of four putative off-target sites

putative sites	TIGR loci	Phsical positions	sequence	Mutations detected
OFF TARGET-1	LOC_Os03g55100	Chr3:31345129..31345150	GATGATGAAGGCCGGGACGAGG	none
OFF TARGET-2	LOC_Os03g05806	Chr3:2901783..2901802	CGCCCCGGACTTCAACATCA	none
OFF TARGET-3	LOC_Os04g03890	Chr4:1780086..1780107	CCCCGCCCCGAACGGCATCATC	none
OFF TARGET-4	LOC_Os07g30210	Chr7:17847716..17847737	GAAGATGAAGACCGGGGCGAGG	none
